# Comparison of Canada-United Kingdom-Australia (CANUKA) scores of patients with gastrointestinal bleeding presenting to the emergency department with other gastrointestinal bleeding scores

**DOI:** 10.55730/1300-0144.6064

**Published:** 2025-09-15

**Authors:** Fatma HANÇER ÇELİK, Necmi BAYKAN, Fatma ÜNLÜ, Mustafa ALPASLAN, Ayşe Şule AKAN, Ömer SALT, İbrahim TOKER, Nuh Mehmet BÜYÜKBERBER

**Affiliations:** 1 Department of Emergency Medicine, Gaziantep City Training and Research Hospital, Gaziantep, Turkiye; 2 Department of Emergency Medicine, Faculty of Medicine, Kayseri City Training and Research Hospital, Kayseri, Turkiye; 3 Department of Emergency Medicine, Nevşehir State Hospital, Nevşehir, Turkiye; 4 Department of Emergency Medicine, Faculty of Medicine, Erzurum City Training and Research Hospital, Erzurum, Turkiye; 5 Division of Gastroenterology, Department of Internal Medicine, Faculty of Medicine, Kayseri City Training and Research Hospital, Kayseri, Turkiye

**Keywords:** Canada-United Kingdom-Australia (CANUKA) Score, AIMS65 Score, Rockall Score, Glasgow-Blatchford Score, gastrointestinal system bleeding, emergency medicine, mortality

## Abstract

**Background/aim:**

Acute gastrointestinal (GI) tract bleeding is a common and potentially life-threatening condition among patients presenting to emergency departments. In this study, we calculated AIMS65, Rockall, Glasgow-Blatchford Score (GBS), and Canada-United Kingdom-Australia (CANUKA) scores in patients with GI bleeding admitted to the emergency department and compared the sensitivity of these scoring systems in predicting the rates of admission to intensive care units and mortality. It is aimed to contribute to clinical practice and help determine an effective risk assessment tool in the management of patients with GI bleeding.

**Materials and methods:**

The study was conducted with patients who were diagnosed with GI bleeding. The study was conducted retrospectively between 1 January 2020 and 31 December 2023. The data of the patients were obtained from the hospital automation system. Patients with missing data were excluded from the study. AIMS65, Rockall, GBS, and CANUKA scores of the patients were calculated and recorded separately.

**Results:**

A total of 916 patients were included in our study. The median age was 70 years, and 62.3% of the patients were male. A total of 22.2% of the patients were hospitalized in the intensive care unit (ICU), and the in-hospital mortality rate was 0.9% (n = 8). According to the results of receiver operator characteristic (ROC) analysis of continuous measurements in terms of ICU hospitalization, the ability of the 4 scores to predict ICU hospitalization was statistically significant (p < 0.001). The CANUKA Score had the highest and best discriminative ability to predict ICU admission (area under the ROC curve [AUC] = 0.734). According to the results of ROC analysis of continuous measures in terms of mortality, the ability of AIMS65, CANUKA, and Rockall scores to predict mortality was statistically significant (p-values <0.001, <0.001, and 0.001, respectively).

**Conclusion:**

The CANUKA Score had the best discriminative ability in predicting intensive care unit admission and the best discriminative ability in predicting mortality after the AIMS65 Score.

## 1. Introduction

Acute gastrointestinal (GI) tract bleeding is a common and potentially life-threatening condition among patients presenting to emergency departments. In the management of these patients, various scoring systems have been developed to predict the risk of mortality and morbidity. These include the AIMS65, Rockall, Glasgow-Blatchford Score (GBS), and the recently proposed Canada-United Kingdom-Australia (CANUKA) scores.

The AIMS65 Score attempts to predict the risk of mortality in patients with GI bleeding and is calculated by evaluating five parameters, including albumin level, International Normalized Ratio (INR) level, mental status level, systolic blood pressure, and age [1]. The Rockall Score is used to predict mortality and the risk of rebleeding and is calculated using factors such as age, signs of shock, comorbidities, endoscopic findings, and bleeding findings [2]. The Glasgow-Blatchford Score helps identify low-risk patients and is calculated by evaluating clinical and laboratory findings such as hemoglobin level, blood urea nitrogen level, systolic blood pressure, pulse, presence of melena, presence of syncope, liver disease, and heart failure. The Glasgow-Blatchford Score has been reported to be effective in predicting the need for transfusion and endoscopic treatment [3]. It is a scoring system developed by Rockall et al. [2] and used to predict mortality and rebleeding risk in patients with GI bleeding. This score evaluates five parameters, including the patient’s age, shock findings, comorbidities, endoscopic findings, and endoscopic appearance of the lesion. High scores are associated with increased mortality and rebleeding risk [4].

The recently proposed CANUKA Score was developed with data obtained from Canada, the United Kingdom, and Australia, and aims to perform risk assessment in patients with GI bleeding. The CANUKA Score also uses the patient’s age, presence of melena, presence of hematemesis, presence of syncope, presence of liver disease or malignancy, pulse rate, systolic blood pressure, hemoglobin, and urea levels. However, there are limited studies in the literature on the efficacy of the CANUKA Score compared with other scoring systems.

In this study, AIMS65, Rockall, GBS, and CANUKA scores were calculated in patients with GI bleeding admitted to the emergency department, and the sensitivity of these scoring systems in predicting the rates of admission to intensive care units and mortality was compared. The study aims to contribute to clinical practice and help determine an effective risk assessment tool for the management of patients with GI bleeding.

## 2. Materials and methods

The study was conducted with patients who were diagnosed with GI bleeding and followed up in the Emergency Medicine Clinic of Kayseri City Hospital. The study was conducted retrospectively between 1 January 2020 and 31 December 2023, and included patients of both sexes aged 18 years and over. The data of the patients were obtained from the hospital automation system. Patients with missing data were excluded from the study. The AIMS65, Rockall, GBS, and CANUKA scores of the patients were calculated and recorded separately. All patient data were obtained from the hospital’s automated system. Because our hospital’s operating principles require integration with the hospital’s automated system, the physical patient files were also integrated, so even though the physical patient files were not physically accessible, the data from the physical files were used. To identify patients with GI bleeding, the patient list was accessed using the International Classification of Diseases, 10th Revision (ICD-10) diagnosis codes hematemesis (K92.0), melena (K92.1), and gastrointestinal hemorrhage (K92.2). Because no other diagnostic codes were assigned to patients with suspected GI bleeding in the secretariat unit, these codes were used for screening. Patients with these diagnostic codes were individually reviewed, and those with incomplete data or without a diagnosis of GI bleeding were excluded. Although these diagnostic codes were obtained for 1145 patients during the study period, 229 patients with missing data and no definitive diagnosis of GI bleeding were excluded, leaving 916 patients for the study. Statistical analyses were performed with SPSS version 22 (IBM Corp., Armonk, NY, USA). The normality assessment of the numerical data recorded in the study was checked by Kolmogorov–Smirnov and Shapiro–Wilk tests. In descriptive statistics, mean ± standard deviation was used for parametric data if they fit the normal distribution, or median (minimum–maximum) if they did not fit the normal distribution, and frequency and percentage values were used for categorical data. In the comparison of parametric measurements between groups, an independent group t-test was used if the groups were normally distributed, and a Mann–Whitney U test was used if the groups were not normally distributed. The chi-square test was used for comparison of categorical variables. For the expression of significance value, p < 0.05 was accepted. Receiver operator characteristic (ROC) analyses were performed for AIMS65, CANUKA, GBS, and Rockall scores regarding ICU admission and mortality. ROC curves for the parameters were also compared. Descriptive statistics included AUC (the area under the ROC curve), sensitivity, specificity, positive predictive value (PPV), negative predictive value (NPV), positive likelihood ratio (LR+), negative likelihood ratio (LR–), and 95% confidence interval (CI). ROC analyses were calculated using MedCalc Statistical Software version 20.305 (MedCalc Software Ltd, Ostend, Belgium; https://www.medcalc.org; 2023) program. The optimum cut-off values were determined using the Youden index.

## 3. Results

Our study included 916 patients who presented to the Emergency Medicine Clinic of Kayseri City Hospital and were diagnosed with GI bleeding. The majority of patients’ endoscopy procedures were performed in the emergency department during the diagnosis and follow-up period, and all of them underwent endoscopy within the first 24 h. The median age of the patients was 70 years (IQR: 55–81, min: 19, max: 111), and 62.3% (n = 571) of the patients were male. 22.2% (n = 203) of the patients were hospitalized in the intensive care unit (ICU), and the in-hospital mortality rate was 0.9% (n = 8).

A statistically significant difference was found between age, AIMS65, CANUKA, GBS, and Rockall scores and ICU hospitalization (p < 0.001). The median values of age, AIMS65, CANUKA, GBS, and Rockall scores of patients hospitalized in the intensive care unit were higher than those of patients hospitalized in the ward. When analyzed in terms of mortality, a statistically significant difference was found between AIMS65, CANUKA, and Rockall scores and mortality (p-values <0.001, 0.002, and 0.019, respectively). The median values of AIMS65, CANUKA, and Rockall scores of the patients who died were higher than those who survived (as shown in Table 1).

**Figure 1:**
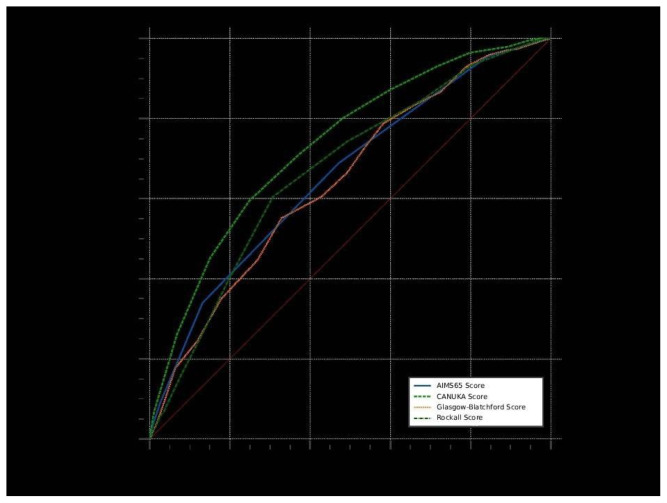
Comparison of ROC curves for predicting ICU admission.

According to the results of ROC analysis of continuous measurements in terms of ICU admission, the ability of the 4 scores to predict ICU admission was statistically significant (p < 0.001). The CANUKA Score had the highest and best discriminative ability to predict ICU admission (AUC = 0.734). A CANUKA Score >9 had a sensitivity of 59.6%, specificity of 75%, positive predictive value of 40.5%, negative predictive value of 86.7%, positive likelihood ratio of 2.39, and negative likelihood ratio of 0.54 in predicting ICU admission. In pairwise comparisons of ROC curves in terms of ICU admission, statistically significant differences in terms of AUC values were found between CANUKA vs. AIMS65 (p < 0.001), CANUKA vs. GBS (p < 0.001), and CANUKA vs. Rockall (p < 0.001) scores (as shown in Table 2, Figure 1).

**Figure 2:**
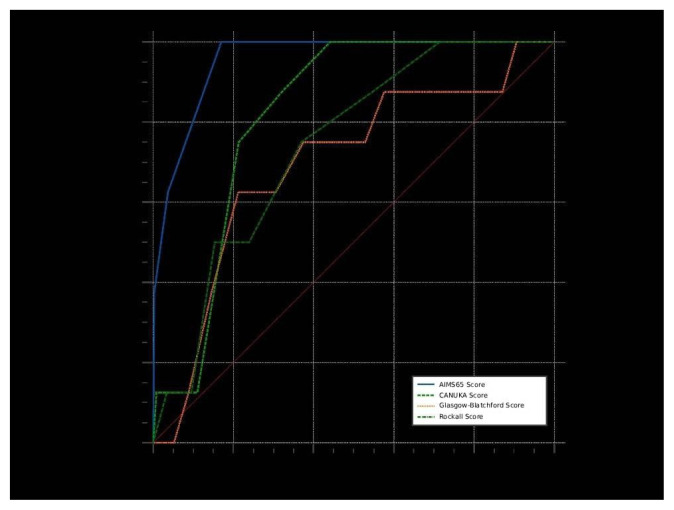
Comparison of ROC curves for predicting mortality by scores.

According to the results of ROC analysis of continuous measures in terms of mortality, the ability of AIMS65, CANUKA, and Rockall scores to predict mortality was statistically significant (p-values <0.001, <0.001, and 0.001, respectively). The AIMS65 Score (AUC = 0.955) had the highest and excellent discriminative ability to predict mortality (p < 0.001). With an AIMS65 Score of >2, the sensitivity was 100%, specificity 82.9%, positive predictive value 4.9%, negative predictive value 100%, positive likelihood ratio 5.86, and negative likelihood ratio 0 for predicting mortality. Pairwise comparisons of ROC curves for mortality showed statistically significant differences in AUC values between AIMS65 vs. CANUKA (p < 0.001), AIMS65 vs. GBS (p = 0.010), and AIMS65 vs. Rockall (p = 0.007) scores (as shown in Table 3, Figure 2).

## 4. Discussion

GI bleeding is a serious clinical condition requiring rapid diagnosis and effective intervention in emergency department visits. Despite advances in intensive care and the development of endoscopic treatment methods, mortality rates in GI diseases are still significant [5]. Since GI bleeding is a condition that may lead to serious complications in the absence of timely intervention, early risk assessment plays a critical role in terms of the appropriate referral of patients. Prognostic scoring systems have the potential to standardize patient-specific risk assessment by providing an objective framework for clinical decision-making. They also facilitate the transfer of information between different clinicians, contributing to continuity and consistency in patient care. Determining which patients are at higher risk of mortality or morbidity is also of great importance for the efficient and prioritized use of limited resources, such as emergency endoscopy or intensive care beds. The initial evaluation and treatment of patients with GI bleeding in the emergency department often have to be initiated before endoscopic findings are obtained. Therefore, some prognostic scoring systems are limited in terms of early applicability because they require endoscopy. In this study, three clinical scoring systems (AIMS65, GBS, and CANUKA) that can be applied without the need for endoscopic data, and the classical Rockall Score, including endoscopic findings, were evaluated. In this context, the clinical predictive power of various multifactorial prognostic scoring models based on both noninvasive and endoscopic data from patients with GI bleeding was analyzed. The aim was to identify the most appropriate scoring systems that can support rapid decision-making and early risk stratification in emergency department settings.

The lower mortality rate in this study compared to other studies could be attributed to the fact that patients did not present to the hospital late, patients were able to undergo early interventional procedures, and the effectiveness of the medical treatment. In this study, the ability of the CANUKA scoring system to predict ICU admission and in-hospital mortality in patients with GI bleeding was compared with the widely used AIMS65, GBS, and Rockall scores. Our findings show that the CANUKA Score has the best discriminatory power in predicting ICU admission, but is second only to the AIMS65 Score in predicting mortality. It is consistent with the literature data that the CANUKA Score may be useful in predicting adverse outcomes and mortality in patients with GI bleeding [6]. It is thought that the use of CANUKA will be advantageous, especially in terms of preventing unnecessary ICU hospitalizations and using hospital resources effectively.

In our study, the GBS score was found to be significant in predicting ICU hospitalization, but did not show a statistically significant difference in predicting mortality. This finding overlaps with the data from a study conducted in 2019 that GBS is more successful in determining bleeding severity and predicting the need for transfusion rather than mortality [7]. Transfusion needs were determined based on current literature data, and patients with indications for transfusion were given red blood cell replacement until the patient reached their target hemoglobin level. According to international validation analyses, the CANUKA Score has a similar accuracy level to the GBS in predicting patient outcomes, but stands out by more accurately identifying patients at low risk for adverse clinical outcomes [8].

In terms of mortality prediction, the AIMS65 Score had the highest AUC value (0.955) and showed excellent discrimination. These results are consistent with the findings in the literature, which revealed that AIMS65 performed better than GBS and Rockall scores in predicting in-hospital mortality [9,10].

In comparative analyses, while the CANUKA Score identifies low-risk patients with high accuracy in terms of mortality and the need for intervention, it draws attention with its higher negative predictive value and sensitivity in predicting intensive care unit admission and short-term mortality compared with the Rockall score [11]. Our study confirms these results and reveals that the CANUKA Score is a more reliable tool than the Rockall Score in both predicting intensive care unit hospitalization and identifying low-risk patients.

In the literature, it has been shown that the CANUKA Score has a higher discriminative power compared with the GBS and modified GBS (mGBS) scores and provides significant superiority in predicting mortality, especially in patients younger than 82 years [7]. These findings are largely compatible with the results of our study.

There is a limited number of comprehensive studies directly comparing the performance of the CANUKA Score with other risk scoring systems in predicting intensive care unit admission and mortality. In particular, studies on the capacity of CANUKA to predict the need for intensive care and its role in identifying low-risk patients have mostly limited sample sizes, and external validation of this score is still insufficient. In this context, our study is one of the rare studies comparing the CANUKA Score with other scoring systems in terms of both mortality and intensive care admission and demonstrating statistically significant differences. We think that the discriminative power of CANUKA in these two critical endpoints makes an important contribution in terms of providing data showing that it can be used in clinical decision-making processes.

According to the findings of our study, the CANUKA Score provides important predictions in terms of both mortality and intensive care hospitalization and stands out as an effective risk assessment tool in clinical decision-making processes. Compared with other scoring systems such as AIMS65, GBS, and Rockall, one of the most important advantages of CANUKA is its ease of calculation. In addition, the fact that it can be applied without the need for endoscopic findings and takes into account clinically critical parameters, such as age, vital signs, and laboratory values of the patient, makes it a practical scoring system, especially in emergency departments and hospitals with limited access to endoscopy. In this respect, the CANUKA Score can contribute to the early identification of high-risk patients not only in advanced centers but also in resource-limited healthcare facilities.

This study shows that the CANUKA Score has the highest discriminative power in predicting ICU admission in patients with GI bleeding, whereas only AIMS65 is superior to CANUKA in predicting mortality. We conclude that in clinical practice, CANUKA can be used as a decision support tool to predict ICU admission, but AIMS65 is more effective in identifying critically ill patients.

## Figures and Tables

**Table 1:** Comparison of age, gender, AIMS65, CANUKA, GBS and Rockall scores in terms of ICU hospitalization and mortality.

	Total n=916	Ward n=713	ICU n=203	p	Survived n=908	Died n=8	p
**Male**, n (%)	571 (62.3)	449 (63.0)	122 (60.1)	0.456*	564 (62.1)	6 (87.5)	0.140*
**Age**, years, median (IQR)	70 (55–81)	70 (52.5–80)	75 (60–83)	**<0.001**	70 (54.2–81)	73.5 (69.5–83.7)	0.223
**AIMS65**, median (IQR)	2 (1–2)	1 (1–2)	2 (1–3)	**<0.001**	2 (1–2)	4 (3–5)	**<0.001**
**CANUKA**, median (IQR)	8 (6–10)	7 (5–9.5)	10 (8–12)	**<0.001**	8 (6–10)	11 (10.2–11)	**0.002**
**GBS**, median (IQR)	10 (6–13)	9 (5–13)	12 (8–14)	**<0.001**	10 (6–13)	14 (9.7–15)	0.058
**Rockall**, median (IQR)	4 (2–5)	3 (2–5)	5 (3–7)	**<0.001**	4 (2–5)	6 (4.2–7)	**0.019**

AIMS65: AIMS65 Score; GBS: Glasgow-Blatchford Score; CANUKA: Canada-United Kingdom-Australia (CANUKA) Score.

* Chi-square test; Other statistical analyses were calculated with the Mann–Whitney U test.

**Table 2:** ROC curve analyses of scores in terms of ICU admission.

		AUC (p-score)	Cut-off	Sensitivity	(%95 CI)	Specificity	(%95 CI)	LR+	LR−	PPV	NPV
ICU	AIMS65	0.658 (<0.001)	>1	68.97	62.1–75.3	52.73	49.0–56.5	1.46	0.59	29.4	85.6
	CANUKA	0.734 (<0.001)	>9	59.61	52.5–66.4	75.04	71.7–78.2	2.39	0.54	40.5	86.7
	GBS	0.644 (<0.001)	>11	55.17	48.1–62.1	67.04	63.5–70.5	1.67	0.67	32.3	84.0
	Rockall	0.669 (<0.001)	>4	60.59	53.5–67.4	69.28	65.8–72.7	1.97	0.57	36.0	86.1

AIMS65: AIMS65 Score; GBS: Glasgow-Blatchford Score; CANUKA: Canada-United Kingdom-Australia (CANUKA) Score.

**Table 3:** ROC curve analyses of scores in terms of hospitalization mortality.

		AUC (p-score)	Cut-off	Sensitivity	(%95 CI)	Specificity	(%95 CI)	LR+	LR−	PPV	NPV
Mortality	AIMS65	0.955 (<0.001)	>2	100.0	63.1–100.0	82.9	80.3–85.3	5.86	0.0	4.9	100.0
	CANUKA	0.817 (<0.001)	>8	100.0	63.1–100.0	55.8	52.5–59.1	2.26	0.0	2.0	100.0
	GBS	0.694 (0.054)	>13	62.5	24.5–91.5	78.7	75.9–81.4	2.94	0.48	2.5	99.6
	Rockall	0.739 (0.001)	>4	75.0	34.9–96.8	63.0	59.8–66.1	2.03	0.40	1.8	99.7

AIMS65: AIMS65 Score; GBS: Glasgow-Blatchford Score; CANUKA: Canada-United Kingdom-Australia (CANUKA) Score.

## References

[1] Kim MS Choi J Shin WC AIMS65 scoring system is comparable to Glasgow-Blatchford score or Rockall score for prediction of clinical outcomes for non-variceal upper gastrointestinal bleeding BMC Gastroenterology 2019 19 1 136 10.1186/s12876-019-1051-8 31349816 PMC6660932

[2] Rockall TA Logan RF Devlin HB Northfield TC Risk assessment after acute upper gastrointestinal haemorrhage Gut 1996 38 3 316 321 10.1136/gut.38.3.316 8675081 PMC1383057

[3] Stanley AJ Dalton HR Blatchford O Ashley D Mowat C Multicentre comparison of the Glasgow Blatchford and Rockall scores in the prediction of clinical end-points after upper gastrointestinal haemorrhage Alimentary Pharmacology and Therapeutics 2011 34 4 470 475 10.1111/j.1365-2036.2011.04747.x 21707681

[4] Tohidi N Movahedi M Rukerd MRZ Mirkamali H Alizadeh SD Comparative analysis of four upper gastrointestinal bleeding scoring systems for predicting multiple outcomes: an observational study in the emergency department Frontiers in Emergency Medicine 2024 8 3 e24 e24 10.18502/fem.v8i3.16331

[5] Liu S Zhang X Walline JH Yu X Zhu H Comparing the performance of the ABC, AIMS65, GBS, and pRS scores in predicting 90-day mortality or rebleeding among emergency department patients with acute upper gastrointestinal bleeding: A prospective multicenter study Journal of Translational Internal Medicine 2021 9 2 114 122 10.2478/jtim-2021-0026 34497750 PMC8386323

[6] Goff S Friedman E Toro B Almonte M Wilson C Utility of the CANUKA scoring system in the risk assessment of upper GI Bleeding Journal of Clinical Gastroenterology 2023 57 6 595 600 10.1097/mcg.0000000000001735 36730919

[7] Di Gioia G Sangineto M Paglia A Cornacchia MG Parente F Limits of pre-endoscopic scoring systems in geriatric patients with upper gastrointestinal bleeding Scientific Reports 2024 14 1 20225 10.1038/s41598-024-70577-2 39215015 PMC11364688

[8] Oakland K Kahan BC Guizzetti L Martel M Bryant RV Development, validation, and comparative assessment of an international scoring system to determine risk of upper gastrointestinal bleeding Clinical Gastroenterology and Hepatology 2019 17 6 1121 1129e2 10.1016/j.cgh.2018.09.039 30268566

[9] Martínez-Cara JG Jiménez-Rosales R Úbeda-Muñoz M de Hierro ML de Teresa J Comparison of AIMS65, Glasgow–Blatchford score, and Rockall score in a European series of patients with upper gastrointestinal bleeding: Performance when predicting in-hospital and delayed mortality United European Gastroenterology Journal 2016 4 3 371 379 10.1177/2050640615604779 27403303 PMC4924428

[10] Gu L Xu F Yuan J Comparison of AIMS65, Glasgow–Blatchford and Rockall scoring approaches in predicting the risk of in-hospital death among emergency hospitalized patients with upper gastrointestinal bleeding: A retrospective observational study in Nanjing, China BMC Gastroenterology 2018 18 1 8 10.1186/s12876-018-0828-5 29954332 PMC6022417

[11] Pognonec C Dirhoussi Z Cury N Moreau M Billard C External validation of Glasgow-Blatchford, modified Glasgow-Blatchford and CANUKA scores to identify low-risk patients with upper gastrointestinal bleeding in emergency departments: a retrospective cohort study Emergency Medicine Journal 2023 40 6 451 457 10.1136/emermed-2022-213052 37185303

